# Chemical Composition, Antifungal and Insecticidal Activities of *Hedychium* Essential Oils

**DOI:** 10.3390/molecules18044308

**Published:** 2013-04-11

**Authors:** Hamidou F. Sakhanokho, Blair J. Sampson, Nurhayat Tabanca, David E. Wedge, Betul Demirci, Kemal Husnu Can Baser, Ulrich R. Bernier, Maia Tsikolia, Natasha M. Agramonte, James J. Becnel, Jian Chen, Kanniah Rajasekaran, James M. Spiers

**Affiliations:** 1 Thad Cochran Southern Horticultural Laboratory, USDA-ARS, 810 Hwy 26 W, Poplarville, MS 39470, USA; E-Mails: Blair.Sampson@ars.usda.gov (B.J.S.); James.Spiers@ars.usda.gov (J.M.S.); 2 National Center for Natural Products Research, The University of Mississippi, MS 38677, USA; E-Mail: ntabanca@olemiss.edu; 3 Natural Products Utilization Research Unit (NPURU), Thad Cochran National Center for Natural Products Research, USDA-ARS, University of Mississippi, MS 38677, USA; E-Mail: dwedge@olemiss.edu; 4 Department of Pharmacognosy, Faculty of Pharmacy, Anadolu University, Eskisehir 26470, Turkey; E-Mails: betuldemirci@gmail.com (B.D.); khcbaser@gmail.com (K.H.C.B.); 5 Botany and Microbiology Department, College of Science, King Saud University, Riyadh 11451, Saudi Arabia; 6 Center for Medical, Agricultural, and Veterinary Entomology (CMAVE), USDA-ARS, 1600 S.W. 23rd Drive, Gainesville, FL 32608, USA; E-Mails: Uli.Bernier@ars.usda.gov (U.R.B.); Maia.Tsikolia@ars.usda.gov (M.T.); Natasha.Elejalde@ars.usda.gov (N.M.A.); James.becnel@ars.usda.gov (J.J.B.); 7 National Biological Control Laboratory, Biological Control of Pests Research Unit, USDA-ARS, 59 Lee Road, Stoneville, MS 38776, USA; E-Mail: jian.chen@ars.usda.gov; 8 Southern Regional Research Center, USDA-ARS, 1100 Robert E. Blvd, New Orleans, LA 70124, USA; E-Mail: Rajah.Rajasekaran@ars.usda.gov

**Keywords:** *Hedychium* cultivars, natural botanical insecticides, azalea lace bugs, yellow fever mosquito, red imported fire ants

## Abstract

The antimicrobial properties of essential oils have been documented, and their use as “biocides” is gaining popularity. The aims of this study were to analyze the chemical composition and assess the biological activities of *Hedychium* essential oils. Oils from 19 *Hedychium* species and cultivars were analyzed by gas chromatography (GC) and gas chromatography-mass spectrometry (GC-MS) techniques. The antifungal and insecticidal activities of these oils were tested against *Colletotrichum acutatum*, *C. fragariae*, and *C. gloeosporioides*, and three insects, the azalea lace bug (*Stephanitis pyrioides*), the yellow fever mosquito (*Aedes aegypti*), and the red imported fire ant (*Solenopsis invicta*). *Hedychium* oils were rich in monoterpenes and sesquiterpenes, especially 1,8-cineole (0.1%–42%), linalool (<0.1%–56%), α-pinene (3%–17%), β-pinene (4%–31%), and (*E*)-nerolidol (0.1%–20%). *Hedychium* oils had no antifungal effect on C. gloeosporioides, C. fragariae, and C. acutatum, but most *Hedychium* oils effectively killed azalea lace bugs. The oils also show promise as an adult mosquito repellent, but they would make rather poor larvicides or adulticides for mosquito control. *Hedychium* oils acted either as a fire ant repellent or attractant, depending on plant genotype and oil concentration.

## 1. Introduction

The genus *Hedychium* Koenig is a member of the Zingiberaceae family and consists of about 80 perennial herbaceous species characterized by showy and scented flowers. They are generally used as ornamentals, but they are also used for their industrial (paper manufacture and perfumery) or medicinal properties [1,2,3] Hedychiums have been reported to possess antibacterial, antifungal, and insecticidal activities [4,5].

Strawberry anthracnose, caused by the plant pathogens *Colletotrichum* species is one of the most important diseases affecting strawberries worldwide [6]. *Colletotrichum fragariae* Brooks is most often associated with anthracnose crown rot of strawberries grown in hot, humid areas such as the southeastern United States [7].

The azalea lace bug [*Stephanitis pyrioides* (Scott)] is a major leaf pest of azalea plants in nurseries and landscapes. Foliar injury to host tissue by this insect is mostly cosmetic and appears as black ovipositional scabs, leaf stippling and leaf chlorosis. However, if there is no early control for lace bug feeding in the landscape, leaf chlorosis induced by the bug’s incessant feeding can progress to necrosis and eventually defoliation and plant death. Azalea lace bugs are difficult to control, as adult females protect each egg by laying them underneath the leaf and sheltering them with a hard fecal dome. Such egg-laying behaviors will minimize an azalea’s exposure to natural enemies, and water-soluble insecticides as well [8,9]. Yellow fever mosquitoes, *Aedes aegypti* L., can transmit pathogens that cause dengue, dengue hemorrhagic fever, yellow fever, and filariasis. This complex of mosquito-borne diseases is second only to malaria in morbidity and mortality on a global scale. Screening of repellents against *Ae. aegypti* is conducted worldwide because this mosquito is globally distributed, and females are anthropophilic (as well as zoophilic), but easily repulsed by odiferous repellents [10]. Red imported fire ants (*Solenopsis invicta* Buren) are a serious pest to humans, wildlife, and crops and cost the US economy over $6 billion annually [11].

Controlling the fungi and insects mentioned above is usually accomplished using traditional fungicides and insecticides, respectively, which could contribute greatly to health, ground water, and environmental problems. Thus, we seek natural botanical fungicides and insecticides that are formulated as flowable emulsions and readily kill or repel insects or pathogens outright, yet are safe for routine use [12,13]. Therefore, 19 different *Hedychium* essential oils were characterized by GC and GC-MS and evaluated for their antifungal and insecticidal potency against *C. acutatum*, *C. fragariae*, *C. gloeosporioides* and three insects, namely the azelaea lace bug (*S. pyrioides*), the yellow fever mosquito (*Ae. aegypti*), and the red imported fire ant (*So. invicta*).

## 2. Results and Discussion

### 2.1. Essential oil Chemical Composition

Nineteen *Hedychium* essential oils were analyzed by GC and GC-MS using a polar column and constituents were compared to known compounds using the in-house Baser Library and MS literature data. Eighty-seven compounds were identified from *Hedychium* oils which constituted 88.6% to 99.0% of the total oil. Identified compounds in *Hedychium* oils with their relative percentages are listed in Table 1. The majority of essential components in *Hedychium* oils were found in the monoterpene constituents where the most prominent compounds were 1,8-cineole (0.1%–42%), linalool (<0.1%–56%), α-pinene (3%–17%), β-pinene (4%–31%) followed by sesquiterpene constituents such as (*E*)-nerolidol (0.1%–20%). The compound 1,8-cineole was, by far, the most ubiquitous constituent of the oils as it was found in 16 out of the 19 *Hedychium* genotypes, and in 13 of those genotypes, 1,8-cineole was the dominant constituent (Table 1). Essential oils from several *Hedychium* species, such as *Hedychium coronarium*, *H. acuminatum*, *H. gardnerianum*, and *H. spicatum* have been investigated [14,15,16,17]. Additionally, among the genotypes included in our study were *Hedychium coccineum*, *H. flavescens* and *H*. *flavum* whose essential oils have been studied phytochemically [18,19]. The major essential oil components of *Hedychium coccineum* grown in Mauritius were (*E*)-nerolidol (44.4%) and *trans*-sesquisabinene hydrate (24.2%) [19]. However, *H. coccineum* oil in our study was dominated by linalool (27%) and α-pinene (14%). Moellenbeck *et al.* [18] reported that the major components in *H. flavum* essential oils were β-pinene (50%) and β-caryophyllene (27%). In contrast, the major constituents found in *H. flavum* essential oils in our study were 1,8-cineole (28.3%) and α-pinene (12.1%). It is worth noting that Moellenbeck *et al.* [18] reported using “aerial parts” of *H. flavum* for essential oil extraction, but it is not known whether these “aerial parts” were leaves or pseudostems or both. In our study, the essential oils investigated were from rhizomes. *H. flavescens* oil was characterized by the major compounds linalool (35%), β-pinene (27%), 1,8-cineole (13%) and these amounts are in agreement with previous studies [19].

**Table 1:** Chemical composition of essential oils from different *Hedychium* species and cultivars.

		*Hedychium* source of compounds (1–19)																			
RRI	Compound	1	2	3	4	5	6	7	8	9	10	11	12	13	14	15	16	17	18	19	IM
		%	%	%	%	%	%	%	%	%	%	%	%	%	%	%	%	%	%	%	
1032	α-Pinene	2.8	11	3.3	6.5	8.9	16.4	9.8	5.3	12.1	9.9	13.6	8.3	16.7	3.6	13.5	9.8	14.3	9.8	4.4	a,b
1035	α-Thujene	0.1	0.4	0.4	0.4	0.4	0.5	0.5	0.2	0.5	0.1	0.5	0.5	0.5	0.1	-	0.6	0.4	0.3	0.4	a
1076	Camphene	0.6	0.8	0.7	1.2	0.6	0.7	0.7	0.5	0.7	0.5	0.9	0.2	3.8	0.2	2.3	1.2	1.1	0.4	1.3	a,b
1093	Hexanal	0.1	0.2	-	-	-	0.1	-	0.2	-	0.1	0.1	-	-	-	-	-	0.1	-	-	a
1118	β-Pinene	3.7	23.9	9.4	16.3	27.9	25.4	17.1	7.9	23.7	31.3	8.3	8.7	13.7	14	7.5	30.5	11.8	26.7	7.3	a,b
1132	Sabinene	0.3	0.6	4.4	0.3	0.2	0.3	0.2	0.1	0.2	0.2	0.8	0.6	0.3	0.2	-	0.3	0.3	0.1	-	a,b
1159	δ-3-Carene	0.2	0.2	-	0.6	-	-	0.2	1.1	0.3	0.2	0.5	0.9	0.3	tr	-	-	0.3	-	-	a
1174	Myrcene	0.4	0.5	0.3	0.8	0.2	0.6	0.4	1.1	0.5	0.6	0.9	1.2	0.9	0.3	-	0.4	0.6	0.3	0.3	a,b
1176	α-Phellandrene	-	-	-	0.5	-	-	-	1.1	0.2	0.2	0.6	0.4	0.3	0.2	-	-	0.2	0.1	0.3	a,b
1188	α-Terpinene	-	-	-	-	-	-	-	-	0.1	0.1	0.1	-	-	0.5	-	-	-	-	-	a,b
1203	Limonene	1.9	2.8	1.6	3.3	2.4	1.5	2.6	2.6	3.1	3	2.6	3.4	-	0.9	1.1	2.8	3	1.8	1.9	a,b
1213	1,8-Cineole	39.4	27.4	22.1	30.1	38.7	5.5	42	26.7	28.3	39.3	17	38	25.5	10.1	0.1	34	24.8	12.9	39.1	a,b
1255	γ-Terpinene	-	-	-	0.1	-	0.7	-	0.1	0.5	0.3	1.6	-	0.5	3.4	-	0.1	0.7	0.5	0.3	a,b
1280	*p*-Cymene	1.9	3.4	5	4	1.1	5.7	3.1	6.3	3.7	1.5	8.9	7.3	5.2	1.4	0.5	1.9	4.7	3	1.9	a,b
1290	Terpinolene	-	-	-	0.1	-	0.1	-	0.2	0.2	0.1	0.2	-	0.1	0.3	-	-	0.1	0.1	0.2	a,b
1398	2-Nonanone	-	0.5	-	0.1	-	-	-	-	0.4	-	-	-	-	-	-	-	-	0.1	-	a
1450	*trans*-Linalool oxide	tr	0.1	-	-	-	0.1	-	-	0.1	-	tr	-	-	0.1	1.8	-	0.1	0.1	-	a
	(*Furanoid*)																				
1478	*cis*-Linalool oxide	0.1	0.1	-	-	-	0.2	-	-	0.1	-	0.1	-	-	0.1	2	-	0.1	0.1	-	a
	(*Furanoid*)																				
1499	α-Campholene aldehyde	-	-	-	-	-	-	-	-	0.1	tr	-	-	-	-	0.4	-	tr	tr	0.2	a
1532	Camphor	0.1	0.1	0.2	0.2	0.1	tr	0.1	-	-	tr	0.1	-	0.9	-	0.2	-	0.1	tr	0.3	a,b
1553	Linalool	11.9	8.8	0.1	0.2	tr	23.8	0.4	0.4	8.9	0.8	19.8	0.7	1	56	26.7	0.1	24.2	35	2.2	a,b
1568	*trans*-α-Bergometene	-	-	0.4	0.5	-	0.1	-	-	-	-	0.2	-	-	-	-	0.5	0.1	-	-	a
1571	*trans-p*-Menth-2-en-1-ol	-	-	-	-	tr	tr	0.1	-	-	0.1	-	-	0.1	-	-	-	-	0.1	-	a
1586	Pinocarvone	-	0.5	0.2	0.1	1.1	0.1	0.4	-	0.2	0.2	-	-	-	-	0.5	0.1	-	0.1	-	a,b
1591	Fenchyl alcohol	-	-	-	-	-	-	-	-	-	-	-	-	-	-	-	-	-	-	0.3	a
1591	Bornyl acetate	0.3	-	-	0.1	-	0.2	-	0.8	-	-	-	-	-	-	8.4	0.1	-	-	-	a,b
1601	Nopinone	-	0.2	-	-	0.2	-	-	-	-	-	-	-	-	-	-	-	-	-	-	a
1611	Terpinen-4-ol	2.6	2.1	7.7	1.5	1.1	2.5	2	0.7	1.5	2.8	1.6	6.1	2.5	2.1	0.1	4.1	1.8	2	4.9	a,b
1612	β-Caryophyllene	1.6	1.2	0.6	5.3	0.2	-	1.5	4	3.1	0.1	1.6	6.3	0.7	-	-	0.2	2.2	0.2	1.2	a,b
1638	*cis-p*-Menth-2-en-1-ol	-	-	0.2	0.1	-	-	-	-	-	0.1	-	-	-	-	-	-	-	-	-	a
1648	Myrtenal	-	0.3	0.3	0.1	0.7	0.1	0.3	-	0.1	0.2	-	-	-	-	0.7	0.2	0.1	-	tr	a
1661	Alloaromadendrene	-	-	-	-	-	1.2	-	-	-	-	0.2	-	0.8	-	-	-	-	-	-	a
1670	*trans*-Pinocarveol	-	0.5	0.7	0.2	1.1	-	0.4	0.1	0.2	0.1	-	0.1	-	-	1.5	0.3	0.1	0.2	0.4	a,b
1682	δ-Terpineol	0.3	0.1	-	0.2	0.3	-	0.2	0.1	0.1	0.3	0.3	0.2	0.2	-	-	0.2	0.1	0.2	0.3	a
1683	*trans*-Verbenol	-	-	-	-	0.3	-	-	-	-	-	-	-	-	-	0.9	-	-	-	-	a,b
1687	α-Humulene	0.3	0.5	0.3	0.5	0.3	tr	0.4	17.6	0.7	0.1	0.1	0.8	0.2	-	-	0.1	0.2	-	0.2	a,b
1706	α-Terpineol	1.7	3.8	1.1	1.6	1.9	1.4	5	1	4.6	4.8	1.2	1.3	6.6	1.1	0.6	5.9	1.3	2.2	5.6	a,b
1709	α-Terpinyl acetate	-	-	-	-	-	-	3	-	-	-	-	0.8	-	-	-	-	-	-	-	a,b
1719	Borneol	2.6	1.4	1.5	2.6	1	0.8	-	0.1	1.2	0.8	2.2	-	4.5	0.3	1	2.4	2	0.4	5.3	a,b
1725	Verbenone	-	-	0.1	-	tr	-	tr	-	-	-	-	-	-	-	0.5	-	-	-	-	a
1729	*cis*-1,2-Epoxy-terpin-4-ol	0.1	-	-	-	0.2	0.2	0.1	-	-	-	-	-	-	-	-	0.1	-	-	-	a
1740	Geranial	0.1	-	0.2	0.3	-	-	-	-	-	-	-	-	-	-	-	-	-	-	-	a
1740	α-Muurolene	-	-	-	-	-	-	-	-	-	-	0.2	-	-	-	-	-	-	-	-	a
1741	β-Bisabolene	-	0.1	-	-	0.2	0.2	-	-	-	-	-	-	-	-	-	0.1	-	-	-	a,b
1750	*cis*-Linalool oxide	-	-	-	-	-	-	-	-	-	-	-	-	-	-	0.3	-	-	-	-	a
	(*Pyranoid*)																				
1755	β-Curcumene	-	-	-	-	-	-	-	-	0.2	-	0.8	-	-	-	-	-	0.2	-	-	a
1770	*trans*-Linalool oxide		-	-	-	-	-	-	-	-	-	-	-	-	-	0.2	-	-	-	-	a
	(*Pyranoid*)																				
1772	Citronellol	0.5	-	0.1	0.7	-	0.2	-	-	-	-	-	0.3	-	-	-	-	-	-	0.2	a,b
1773	δ-Cadinene	-	-	-	-	-	0.3	-	-	0.2	-	0.8	-	0.2	-	-	-	0.1	tr	0.2	a
1776	γ-Cadinene	-	-	-	-	-	0.1	-	-	0.1	-	0.2	-	0.1	-	-	-	tr	-	0.2	a
1786	*ar*-Curcumene	0.8	2.5	0.1	0.1	-	-	0.1	0.1	1.4	-	4.2	1	1.2	-	4.1	0.2	1.6	-	-	a
1804	Myrtenol	0.1	0.3	0.3	0.1	0.9	0.1	0.3	-	0.1	0.1	-	-	-	-	1.2	0.2	tr	0.1	0.3	a,b
1823	*p*-Mentha-1(7),5-dien-2-ol	-	-	-	0.1	-	-	-	0.3	-	-	0.2	0.2	-	-	-	-	0.1	-	-	a
1823	Cabreuva oxide-VI	-	-	-	-	-	-	-	-	-	-	-	-	-	-	-	-	-	-	2.9	a
1845	*trans*-Carveol	-	-	tr	-	-	-	-	-	-	-	-	-	-	-	0.3	tr	tr	-	-	a,b
1857	Geraniol	0.2	-	0.1	0.6	-	0.2	-	-	-	-	-	-	-	0.2	-	-	0.1	-	0.2	a,b
1864	*p*-Cymen-8-ol	tr	-	0.1	tr	-	-	-	-	-	-	-	-	-	-	-	0.1	tr	tr	0.3	a,b
1949	(Z)-3-Hexenyl nonanoate		-	-	-	-	-	-	-	-	-	-	-	-	-	0.8	-	0.1	tr	-	a
2001	Isocaryophyllene oxide	0.4	0.3	1.8	0.3	0.6	-	0.6	0.1	-	-	0.1	0.6	-	-	-	-	-	-	-	a
2008	Caryophyllene oxide	2.6	2.1	9.5	2	3	-	4.7	3	0.9	0.2	0.6	4.4	0.4	-	1.5	0.5	0.6	tr	3.8	a,b
2045	Humulene epoxide-I	-	0.1	-	-	0.1	-	-	4.5	0.1	-	-	-	-	-	-	-	-	-	-	a
2050	(*E*)-Nerolidol	18.8	-	19.5	13.7	0.8	6.3	0.5	0.2	-	-	0.2	2.1	4.6	1.1	4.6	1.3	0.1	-	3.6	a,b
2071	Humulene epoxide-II	0.2	0.3	0.7	0.1	1.4	-	0.3	7.1	0.1	-	0.2	0.3	-	-	-	tr	-	-	0.3	a
2081	Humulene epoxide-III	-	0.1	-	-	0.1	-	-	2.4	0.1	-	-	-	-	-	-	-	-	-	-	a
2098	Globulol	-	-	-	-	-	0.1	-	-	-	-	-	-	-	-	0.8	-	-	-	-	a
2104	Viridiflorol	-	-	-	-	-	0.1	-	-	-	-	-	-	-	-	0.5	-	-	-	-	a
2144	Spathulenol	0.2	0.1	-	-	-	1.1	-	0.3	0.1	-	0.4	-	0.8	0.1	3.1	-	-	-	-	a,b
2170	β-Bisabolol	-	0.1	-	-	-	-	-	-	0.1	-	0.3	-	0.1	-	-	0.1	tr	-	-	a,b
2185	γ-Eudesmol	-	-	-	-	-	-	-	0.3	-	-	-	-	-	-	-	-	-	-	-	a
2187	T-Cadinol	-	tr	-	-	-	0.1	-	-	-	-	0.3	-	0.1	-	-	-	-	-	0.8	a
2209	T-Muurolol	-	tr	-	-	-	0.1	-	-	0.1	-	0.4	-	0.1	-	0.1	-	tr	-	1.1	a
2214	ar-Turmerol	-	-	-	-	-	-	-	-	-	-	-	-	-	-	0.2	-	-	-	-	a
2255	α-Cadinol	-	0.1	-	-	-	0.2	-	-	-	-	0.9	-	0.2	-	0.4	-	0.1	-	2.6	a
2257	β-Eudesmol	-	-	-	-	0.1	-	-	0.1	-	-	-	-	-	-	-	-	-	-	-	a
2269	Guaia-6,10(14)-dien-4β-ol	0.3	0.1	-	-	0.2	-	-	-	0.1	0.1	0.2	-	0.2	-	-	-	tr	0.3	-	a
2316	Caryophylla-2(12),6(13)-dien-5β-ol	-	-	-	-	-	-	-	0.1	tr	-	-	0.1	-	-	-	-	-	-	-	a
	(= *Caryophylladienol I*)																				
2324	Caryophylla-2(12),6(13)-dien-5α-ol	-	-	-	-	-	-	-	-	-	-	-	-	-	-	-	-	-	-	0.4	a
	(= *Caryophylladienol II*)																				
2389	Caryophylla-2(12),6-dien-5α-ol (= *Caryophyllenol I*)	-	-	0.2	tr	tr	-	tr	0.3	-	-	-	-	-	-	-	-	-	-	0.6	a
2392	Caryophylla-2(12),6-dien-5β-ol (= *Caryophyllenol II*)	-	-	0.2	-	-	-	-	0.2	tr	-	-	-	-	-	-	-	-	-	0.8	a
2551	Geranyl linalool	0.3		-	0.1	-	0.3	0.1	0.1	-	-	-	0.1	0.2	0.1	-	-	-	-	-	a
	Total	97.5	97.6	93.4	95.6	96.3	97.6	97.1	97.3	99	98.2	94	94.9	93.5	96.4	88.4	98.4	97.8	97.1	96.6	

**1**: Tai Conch Pink; **2**: *Hedychium thyrsiforme*; **3**: Dave Case; **4**: Pink V; **5**: White Starburst; **6**: *Hedychium elatum*; **7**: Dr Moy; **8**: Pink Sparks; **9**: *Hedychium flavum*; **10**: *Hedychium bousigonianum*; **11**: Tai Monarch; **12**: Tai Empress; **13**: Tai Emperor; **14**: *Hedychium forrestii*; **15***: Hedychium coccineum*; **16**: Kinkaku; **17**: Tai Mammoth; **18**: *Hedychium flavescens*; **19**: Tai Golden Goddess. RRI: Relative retention indices calculated against *n*-alkanes % calculated from FID data; tr = trace (<0.1%). IM = Identification Method. **a**: comparison of mass spectra with the Wiley and Mass Finder libraries and retention times. **b**: comparison with genuine compounds on the HP Innowax column.

Differences in oil constituents from the same genotype or species are not unusual as chemical composition of these oils can vary depending on factors such as genotype, season, and environment. For example, Zheljazkov *et al.* [20] reported differences in oil constituents of the same cultivars from *Ocimum basillicum* L. and *O. sanctum* L. grown in two different locations and attributed these differences to the different environmental conditions such as temperature, soil characteristics, and production system at the two locations of their study. Furthermore, production of a particular type of oil is highly influenced by the physiology of plant, which undergoes, for example, crucial changes during the transition from the vegetative stage to flowering [21,22]. Botanically, *H. flavum* and *H. flavescens* are very close and often confused [23]. In our experiment, the essential oils from these two species were extracted from plants grown under identical environmental conditions in the same greenhouse, but their major essential oil constituents were quantitatively different, *i.e.*, linalool (35% and 8.9%) and 1,8-cineole (12.9% and 28.3%) for *H. flavescens* and *H. flavum*, respectively. Therefore, determination of *Hedychium* essential oils, in conjunction with morphological and genetic characteristics, could be useful in phylogenetic studies of this genus. For the four remaining species, the dominant constituents were β-pinene for *H. elatum* (25.4%) and *H. forrestii* (14%) and 1,8-cineole for *H. thyrsiforme* (27.4%) and *H. bousigonianum* (39.3%). 1,8-Cineole was the dominant component of the oils in the 12 cultivars studied, with the exception of Tai Monarch and Tai Emperor, for which linalool (19.8%) and α-pinene (16.7%) were the major constituents, respectively. This study is the first report on the composition of the essential oils for the majority of the genotypes as well as their antifungal and insecticidal activities against the fungi *C. acutatum*, *C. fragariae*, *C. gloeosporioides* and the insects *St. pyrioides*, *Ae. aegypti* and *So. invicta.*

### 2.2. Antifungal Effects of Hedychium Oils

*Hedychium* oils evaluated for antifungal activity against three *Colletotrichum* species, *C. gloeosporioides*, *C. fragariae*, *and C. acutatum* using direct-bioautography assays showed no antifungal activity at 80, 160 and 320 μg/spots.

### 2.3. Azalea Lace Bug Bioassays

**Figure 1:**
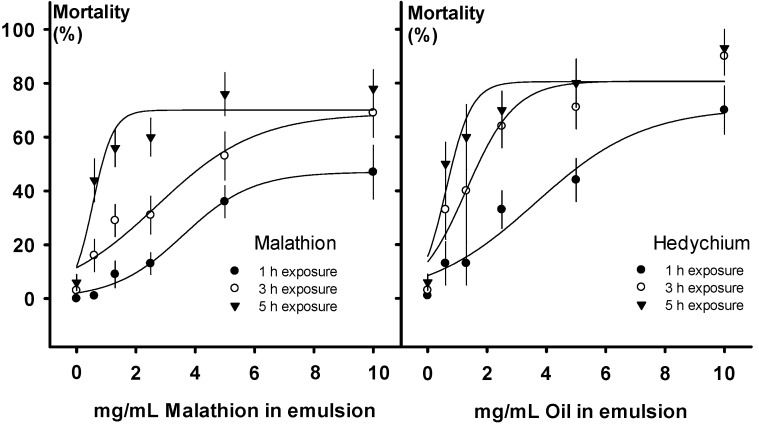
Percentage mortality for adult azalea lace bugs, *Stephanitis pyrioides* (Scott) after they were exposed for 1, 3, and 5 h to six concentrations of *Hedychium* oil or malathion (0.0, 0.6, 1.3, 2.5, 5.0, and 10.0 mg/mL).

*Hedychium* oil was more lethal to azalea lace bugs than the organophosphate insecticide malathion (Chi-square = 47.17, *p* < 0.0001). Approximately, 3–10 mg/mL of *Hedychium* oil or a higher concentration of malathion would be required to kill 80% of bugs (Chi-square = 157.83, *p* < 0.0001) within 5 h of exposure (Figure 1, Chi-square = 117.02, *p* < 0.0001). The comparison of *Hedychium* and malathion required combining all 17 essential oil sources listed in Table 2. Among these, however, eight sources (the first eight listed in Table 2) contributed the most to the potency of *Hedychium* oil, at least when individual oil sources were each tested at 10 mg/mL. The genotypes with the most insecticidal activity were *H. forrestii*, Dave Case, *H. elatum*, *H. bousigonianum*, Tai Mammoth, Tai Conch Pink, Dr. Moy, and Kinkaku. The remaining *Hedychium* essential oils were equivalent to malathion in potency. Only Tai Golden Goddess and the DMSO control (emulsion) had significantly lower insecticidal activity than that of malathion (Table 2). For practical reasons, we had to combine several sources of *Hedychium* oil to obtain enough compound to test against our industrial standard, malathion. Oils extracted from most *Hedychium* species and cultivars were comparable in potency, which is why we decided to mix them into a single concoction. Numerous sources of wildcrafted biomass would probably have to be combined during the commercial production of a botanical pesticide. Therefore, it seems likely that reliably effective oils from sundry *Hedychium* sources would also be similarly used in formulating a product. 

**Table 2:** Mortality of adult azalea lace bugs, *Stephanitis pyrioides* (Scott), to 17 *Hedychium* essential oil extracts and one commercial insecticide (Malathion).All oils from each species or cultivar were compared at a single concentration of 10 mg·L^−1^.

	Activity ^a^				% mortality			
		Ranking		Slope	Exposure time			
Sample oil source		(*p* < 0.05)	*n* (bioassays)		1 h	3 h	5 h	*p*
*Hedychium forrestii*		3	5	0.26	100	100	100	0.003
Dave Case		3	5	0.25	93	100	100	0.005
*Hedychium elatum*		3	5	0.25	93	100	100	0.005
*Hedychium bousigonianum*		3	5	0.23	93	93	93	0.010
Tai Mammoth		3	5	0.22	87	93	93	0.012
Tai Conch pink		3	5	0.22	86	93	93	0.012
Dr. Moy		3	5	0.21	87	87	93	0.019
Kinkaku		3	5	0.20	80	87	93	0.027
Tai Monarch		2	5	0.16	73	87	93	0.062
Tai Empress		2	5	0.15	60	80	93	0.078
*Hedychium flavum*		2	5	0.13	60	80	87	0.127
Tai Emperor		2	5	0.13	73	73	87	0.141
*Hedychium thysiforme*		2	5	0.08	54	80	93	0.350
Pink V		2	5	0.05	60	73	87	0.557
White Starburst		2	5	−0.02	47	73	87	0.849
Pink Sparks		2	5	−0.03	73	73	93	0.719
Tai Golden Goddess		1	5	−0.53	17	42	58	<0.0001
Malathion (baseline)		2	12	0.00	47	69	78	---
DMSO (blank)		0	21	−1.59	1	3	6	<0.0001
OTHER EFFECTS								
exposure time		.		4.50	.	.	.	<0.0001

^a^ Rank indicates level of insecticidal activity relative to the baseline (malathion) at a concentration of 10 mg/mL (*p* < 0.05); *n* = 2,500 adult lace bugs tested; SEM values for *Hedychium* species and cultivars range from 0.06 to 0.09; SEM for Malathion (baseline) = 0.50.

### 2.4. Mosquito Repellency Bioassays

The most potent mosquito repellent oils were obtained from Dave Case (0.129 ± 0.035) mg/cm^2^, followed by Pink V (0.141 ± 0.027) mg/cm^2^, *H. flavescens* (0.164 ± 0.023) mg/cm^2^, *H. thyrsiforme* (0.176 ± 0.073) mg/cm^2^, and Tai Conch Pink (0.187 ± 0.000) mg/cm^2^ (Table 3). The least repellent oils were obtained from Tai Emperor where three out of four volunteers passed using the high dose concentration of 0.375 mg/cm^2^, followed by Tai Monarch (0.328 ± 0.047) mg/cm^2^, and *H. forrestii* (0.328 ± 0.047) mg/cm^2^. For Tai Monarch and *H. forrestii*, three of the four volunteers achieved the required repellency threshold at the highest concentration (0.375 mg/cm^2^) but not at the next lower concentration of 0.187 mg/cm^2^. Oils from Pink Sparks, *H. bousigonianum*, *H. coccineum*, and Tai Golden Goddess were not available in sufficient quantity to determine the minimum effective dosage (MED).

**Table 3:** Minimum effective dosage (MED) of oils and individual constituents tested against the yellow fever mosquito, *Aedes aegypti* (L.), to 17 essential oil extracts and one commercial repellent (DEET). All oils and compounds were evaluated by four volunteers at the highest concentration available and at serial two-fold dilutions until they failed to repel.

Essential oil orCompound	MED (±SEM)(mg/cm^2^) ^a^^,^^b^	Highest Concentration Available (mg/cm^2^)	Range of Repellency (mg/cm^2^) Notes ^c^
Tai Conch Pink	0.187 (0.000)	0.750	0.187–0.187
*Hedychium* *thyrsiforme*	0.176 (0.073)	0.750	0.047–0.375
Dave Case	0.129 (0.035)	0.375	0.047–0.187
Pink V	0.141 (0.027)	0.375	0.094–0.187
White Starburst	0.235 (0.099)	0.375	(nr–2) 0.094–0.375
*Hedychium elatum*	0.235 (0.081)	0.750	0.094–0.375
Dr. Moy	0.234 (0.047)	0.750	0.187–0.375
*Hedychium flavum*	0.258 (0.070)	0.750	0.094–0.375
Tai Monarch	0.328 (0.047)	0.375	0.187–0.375
Tai Empress	0.250 (0.054)	0.375	(nr–1) 0.187–0.375
Tai Emperor	0.375 (0.000)	0.375	(nr–1) 0.375–0.375
*Hedychium forrestii*	0.328 (0.047)	0.375	0.187–0.375
Kinkaku	0.281 (0.54)	0.375	0.187–0.375
Tai Mammoth	0.281 (0.54)	0.375	0.187–0.375
*Hedychium flavescens*	0.164 (0.23)	0.187	0.094–0.187
1,-cineole	0.500 (0.125)	1.500	0.375–0.750
(−)-β-pinene	0.140 (0.047)	1.500	0.047–0.187
(-)-linalool	0.125 (0.031)	1.500	0.094–0.187
(+)-terpinen-4-ol	0.086 (0.051)	1.500	0.023–0.187
(−)-terpinen-4-ol	0.109 (0.041)	1.500	0.047–0.187
α-terpineol	0.039 (0.008)	1.500	0.023–0.047
β-bisabolol	0.035 (0.010)	1.500	0.023–0.047
DEET	0.006 (0.001)	1.500	0.005–0.011

^a^ MED values are reported as the average and standard error of the mean from repellency assays conducted with four volunteers (three male, one female); ^b^ nt indicates for highest dose available indicates that insufficient quantities were available to conduct the repellency assay; ^c^ nr indicates the compound is not repellent at the highest dose tested, the following number indicates the number out of 4 volunteers for which the compound failed to provide repellency at the highest dose.

Some of the major and minor constituents in these oils have also undergone preliminary tests to determine their mosquito repellency (Table 3). One of the major constituents (1,8-cineole) was a less potent repellent (0.500 ± 0.125) mg/cm^2^ than all of the oils that demonstrated repellency and, therefore, not likely to factor into the observed repellency of the oils. Another abundant compound, β-pinene had a MED of (0.140 ± 0.047) mg/cm^2^, which is lower than the MED of most of the oils. Therefore, this compound may play a role, ranging from minor to synergistic, in the oil repellency. Clearly other compounds are contributing to the repellency. The (−)-linalool enantiomer was repellent (0.125 ± 0.031) mg/cm^2^, but this is not totally unexpected since it is a known volatile attraction-inhibitor [24], which produces a volatile masking effect unlike that of the topical or contact effect of the well known repellent *N*,*N*-diethyl-3-benzamide (DEET) [25,26]. In these studies, the MED of DEET was (0.006 ± 0.001) mg/cm^2^.

Trace level constituents such as (+)-terpinen-4-ol (0.086 ± 0.051) mg/cm^2^, and (−)-terpinen-4-ol (0.109 ± 0.041) mg/cm^2^ also showed efficacy that indicates that these may be contributors to the repellency of the oils. Interestingly, of the oil constituents that were assayed for repellency, it was some of the least abundant compounds that produced the highest repellency. The compound α-terpineol, present at <5% and β-bisabolol at <0.3% were determined to have MEDs of (0.039 ± 0.008) mg/cm^2^ and (0.035 ± 0.010) mg/cm^2^, respectively.

### 2.5. Mosquito Larvicidal and Adult Topical Bioassays

In an effort to identify novel classes of plant natural products with activity against *Ae. aegypti*, a high-throughput larval screening method [27] was performed on *Hedychium* essential oils (Table 4). Of the tested oils, most gave 100% mortality at 500 and 250 mg/L. *Hedycium* oils from *H. coccineium*, Kinkaku and Tai Golden Goddess were the most active oils at 125 mg/L. Based on the weak activity found in the larval assays, the whole essential oils were not considered suitable for further testing. *H. flavum* was the most active, but there was no adult mortality observed for these oils at the screening rate of 3.125 mg/L per insect. The results described here for *Hedychium* oils indicated that they have low potential as direct acting toxicity for mosquito control.

### 2.6. Fire Ants

When an extract or compound had digging suppression index (DSI) greater than zero and *p*-value in paired *t*-test less than 0.05, it was claimed as an ant repellent (Supplementary Table S1). *H. thyrsiforme* was demonstrated as a repellent at 100.0 mg/kg. The cultivar Dr. Moy was a repellent at 1.0 and 10.0 mg/kg, but *p*-value at 100 mg/kg for this cultivar was 0.0561, so it failed to be claimed as a repellent. Kinkaku was a repellent at 1.0 mg/kg, but not at 10.0 and 100.0 mg/kg.

When DSI was less than zero and *P*-value less than 0.05, an extract or compound were claimed as an attractant. The cultivar Dave Case was an attractant at 1.0, 10.0 and 100.0 mg/L; Pink V, White Starburst and *H. elatum* at 1.0 and 10.0 mg/kg; *H. flavum* and *H. flavesens* at 100.0 mg/kg; *H. bousigonianum* at 10.0 mg/kg; Tai Empress at 10.0 and 100.0 mg/kg; Tai Emperor, *H. coccineum* at 1.0 mg/kg; *H. forrestii* at 1.0 and 100.0 mg/kg. 

**Table 4:** Larvicidal activities of *Hedycium* essential oils against first instar larvae of *Aedes aegypti.*

Sample oil source	Mortality (%)				
	500 mg/L	250 mg/L	125 mg/L	62.5 mg/L	31.25 mg/L
Tai Conch Pink	100	100	0	0	0
*H. thyrsiforme*	100	100	40	0	0
Dave Case	100	100	80	0	0
Pink V	100	100	60	0	0
White Starburst	100	100	40	0	0
*H. elatum*	100	100	20	0	0
Dr. Moy	100	100	40	0	0
Pink Sparks	100	100	60	0	0
*H. flavum*	100	100	60	40	0
*H. bousigonianum*	100	100	60	0	0
Tai Monarch	100	80	60	0	0
Tai Empress	100	100	40	0	0
Tai Emperor	100	100	80	0	0
*H. forrestii*	100	100	0	0	0
*H. coccineum*	100	100	100	0	0
Kinkaku	100	100	100	0	0
Tai Mammoth	100	100	40	0	0
*H. flavescens*	100	100	60	0	0
Tai Golden Goddess	100	100	100	0	0

Repellency of some natural products has been tested against the red imported fire ants, such as mint oil, and water suspensions of pine needle and cedar shaving [28,29]. Callicarpenal and intermedeol, two terpenoids isolated from the leaves of American beautyberry (*Callicarpa americana* L., Verbenaceae) and Japanese beautyberry (*Callicarpa japonica* Thunb.) were found to be repellent against imported fire ants [30]. An over-the-counter essential oil product in China was found to be a repellent against the workers of red imported fire ants [30]. In this study, the cultivar Pink Sparks showed consistent repellency at all three tested concentrations. The cultivar Dr. Moy was a repellent at 1.0 and 10.0 mg/kg, but not at 100 mg/kg. The cultivar Kinkaku was a repellent at 1.0 mg/kg, but not at 10.0 and 100.0 mg/L. It is hard to explain why repellency was reduced in the cultivars Dr. Moy and Kinkaku with the increase of concentration. However, since we were dealing with an extract, multiple compounds exist in the extract. Some compounds may function as repellents and some as attractants. The result of the bioassay was the combined effect of each compound in the extract. If attractants and repellants in the extract have very different effect-dose curves, the observed “peculiar” results are theoretically possible. For example, if repellants in an extract show their effect at all three concentrations (1.0, 10.0, 100.0 mg/kg), but attractants show their effect only at concentrations above 1.0 mg/L (10.0 and 100 mg/kg), in that situation, we may observe repellency only at 1.0 mg/kg, because the repellency at 10.0 and 100.0 mg/kg may be offset by the attractant effect.

In Chen’s study [30], 1,8-cineole (eucalyptol) at 10 mg/kg showed significant digging facilitation, indicating it was an attractant at that concentration. In this study, Dave Case, Pink’, White Starburst, *H. elatum*, *H. flavum*, *H. bousigonianum*, Tai Empress, Tai Emperor, *H. forrestii*, *H. coccineum*, and *H. flavesens* attracted ants at one or more concentration levels (Supplementary Table S1). Since 1,8-cineole was the most prominent compound found in essential components in *Hedychium* oils (0.1%–42%), it might be reason why *Hedychium* extracts attracted red imported fire ants. However, since no information is available about attractiveness of other monoterpenes and sesquiterpenes found in *Hedychium* oils, their contribution to the attractiveness is unknown. This study has demonstrated that natural products may be an excellent source of fire ant attractants. 

## 3. Experimental

### 3.1. Plant Materials and Isolation of the Essential Oils

Plant materials, obtained from the USDA-ARS ornamental breeding program in Poplarville, Mississippi, are listed in Table 1 and consisted of seven *Hedychium* species, *H. forrestii*, *H. elatum*, *H. bousigonianum*, *H. flavum*, *H. thyrsiforme*, *H. coccineum*, and *H. flavesens* and 12 cultivars, Dave Case, Tai Mammoth, Tai Conch Pink, Dr. Moy, Kinkaku, Tai Monarch, Tai Empress, Tai Emperor, Pink V, White Starburst, Pink Sparks, and Tai Golden Goddess. Most of the species are native to central and southeastern Asia, where they can be found growing in the wild. Voucher specimens of these plants were deposited at the Herbarium of the Faculty of Pharmacy, Anadolu University, Eskisehir, Turkey. Voucher specimen numbers were as follows: Tai Conch Pink (No: 001), *Hedychium thyrsiforme* (No: 002) Dave Case (No: 003), Pink V (No: 004), White Starburst (No: 005), *Hedychium elatum* (No: 006), Dr Moy: (No: 007), Pink Sparks (No: 008), *Hedychium flavum* (No: 009), *Hedychium bousigonianum* (No: 010), Tai Monarch (No: 011), Tai Empress (No: 012), Tai Emperor (No: 013), *Hedychium forrestii* (No: 014), *Hedychium coccineum* (No: 015), Kinkaku (No: 016), Tai Mammoth (No: 017), *Hedychium flavescens* (No: 018), Tai Golden Goddess (No: 019). Essential oils from crushed air-dried *Hedychium* rhizomes were extracted in water using a Clevenger-type distillation apparatus for 3 h. Percentage of oil yield was then calculated on a moisture-free basis (range = 0.08%–0.53%). *Hedychium* essential oils were stored in airtight containers in a refrigerator at 4 °C for subsequent experiments.

### 3.2. Gas Chromatography Analysis Conditions

Oils were analyzed by GC using a Hewlett Packard (SEM Ltd, Istanbul, Turkey) 6890 system equipped with a Flame Ionization Detector (FID). An HP Innowax FSC column (60 m × 0.25 mm *i.d*., 0.25 µm film thickness) was used with nitrogen at 1 mL/min. Oven temperature was set initially at 60 °C and held for 10 min, ramped to 220 °C at 4 °C/min, and held at 220 °C for 10 min, then ramped to 240 °C at 1 °C/min. The oil was injected at a 20% concentration in *n-*hexane. Injector temperature was set to 250 °C. Percentage composition of individual components was obtained from electronic integration using peak areas from chromatographic data of samples analyzed by Gas Chromatography-Flame Ionization Detection (GC-FID, with the FID set to 250 °C). *n*-Alkanes (C9-C20) were used as reference points in the calculation of retention indices (I) [31,32].

### 3.3. Gas Chromatography/Mass Spectrometry (GC/MS) Analysis

GC/MS analysis was performed with a Hewlett-Packard GCD, system (SEM Ltd, Istanbul, Turkey) and Innowax FSC column (60 m × 0.25 mm, 0.25 µm film thickness) was used with helium as carrier gas. GC oven temperature conditions were identical as described above with split flow adjusted to 50 mL/min and injector temperature set at 250 °C. Mass spectra were recorded with 70 eV electrons. Mass range was *m/z* 35 to 425 at a scan rate of 3.46 scans/s. Identification of essential oil components was carried out by comparison of their relative retention times with those of authentic samples or by comparison of their relative retention index (I) to series of *n*-alkanes. Computer matching against commercial (Wiley, Adams and MassFinder 2.1) [33,34,35] and in-house “Baser Library of Essential Oil Constituents” built up by genuine compounds and components of known oils, as well as MS literature data [36,37,38] were also used for identification. 

### 3.4. Fungal Bioassays against Colletotrichum Species

The phytopathogens *Colletotrichum acutatum* Simmonds, *C. fragariae* Brooks, and *C. gloeosporioides* (Penz.) Penz & Sacc. in Penz. were used as target species in our antifungal assay using direct bioautography. Isolates of *C. acutatum*, *C. fragariae* and *C. gloeosporioides* were obtained from Barbara J. Smith (Agricultural Research Service, U.S. Department of Agriculture, Poplarville, MS, USA). Pathogen production and bioautography procedures of Wedge *et al.* [39] were used to evaluate antifungal activity against fungal plant pathogens. Sensitivity of each fungal species to each test compound was determined 4 day after treatment by comparing size of inhibitory zones. Means and standard deviations of inhibitory zone size were used to evaluate antifungal activity of fractions and test compounds. Technical grade commercial fungicides benomyl, cyprodinil, azoxystrobin, and captan (Chem Service, Inc. West Chester, PA, USA) were used as fungicide standards at 2 mM in 2 µL of 95% ethanol. Matrix bioautography was used to screen 19 *Hedychium* essential oils multiple times using both dose- and non-dose-response formats. Antifungal activity can be visualized directly on a TLC plate as ‘clear zones’ free of fungal mycelia, stroma, or condia [40]. Zones with ‘diffuse inhibition’ are considered ‘growth suppressive’ in nature and mycelia, stroma or condia growth occurs at a reduced level. Fungal growth inhibition means for *Hedycium* essential oils were analyzed separately by ANOVA using SAS software, Version 8. Mean separations were performed based on Fisher’s Protected Least Significant Difference (LSD) (*p* = 0.05). Statistical comparisons were made for fungal growth across compounds and of compound across fungal growth.

### 3.5. Azalea Lace Bug Bioassays

Azalea lace bugs (*S. pyrioides*) were reared on bouquets of azalea terminals (*Rhododendron* species) kept in plant growth chambers (Percival Scientific, Perry, IA, USA) at 27 °C, 65% RH with a photoperiod of 14 h/light and 10 h/dark. Three adult lace bugs were transferred to each of 5 to 8 wells per treatment in 96-well microtiter plates. Emulsions of malathion (positive control: *n* = 9 replicated trials) and purified essential oils from 12 cultivars and 5 species of *Hedychium* (Table 2) were each diluted to 10 mg/mL by adding 9% dimethylsulfoxide (DMSO emulsifier) and 90% de-ionized water. *Hedychium* oils bioactivity was tested 5 times for Tai Conch Pink, Dave Case, Dr. Moy, Tai Emperor, Tai Empress, Tai Golden Goddess, Kinkaku, Tai Mammoth, Tai Monarch, Pink V, Pink Sparks and White Starburst. We also tested oils from 5 *Hedychium* species, *H. bousigonianum*, *H. elatum*, *H. flavum*, *H. forrestii*, and *H. thyrsiforme*. The 10 mg/mL dosage was deemed to be the most effective dosage for quickly killing small insects such as bugs and aphids [12,13]. Trace amounts of pure, undiluted oils from all samples were combined to yield enough *Hedychium* oil for testing at five concentrations: 0.06, 0.13, 0.25, 0.50 and 1.00 mg/mL. Our appropriate baseline control or blank for these trials was a 10% aqueous solution of DMSO. Bioassays followed a randomized complete block design (RCBD), with 20 µL of each oil emulsion and baseline solution being pipetted into plastic wells. At the bottom of each well was an absorbent disc of Whatman no. 2 filter paper, which prevented bugs from drowning in residual fluid. We observed adult bugs under a dissecting microscope at the top of every hour for 5 h at 21 °C, to see if any had died. Between these inspections, we kept bugs at 23 °C in a separate growth chamber. The probit analyses, log_e_-transformations of mortality data, and ANCOVA analyses were similar to those used in previous bioassays [12,13].

### 3.6. Mosquito Repellent Bioassays

Mosquitoes used for testing were *Aedes aegypti* (Orlando strain, 1952) from a colony maintained at USDA-ARS-CMAVE in Gainesville, FL. Pupae were removed from the colony and maintained on sugar and water and in laboratory cages at an ambient temperature of 28 ± 1 °C and relative humidity of 35%–60%. Nulliparous mosquitoes were preselected from stock cages using a hand-draw box and trapped in a collection trap [41]. After 500 (±10%) mosquitoes were collected in the trap, they were transferred to a test cage (approximately 59,000 cm^3^ with dimensions 45 cm × 37.5 cm × 35 cm) and allowed to acclimate for 17.5 (±2.5) min before starting a test [42]. Appropriate masses of each candidate repellent were dissolved in 1 mL acteone in a 2-dram vial such that the resulting solution produced a known (mg/cm^2^) application of each compound onto a 50 cm^2^ muslin cloth piece had been inserted into the vial. Vials were sealed and stored at −4 °C until used for the tests, and normally this consisted of <48 h storage time. Prior to the start of testing, the cloth was removed from the vial and affixed by staples onto two sections of card stock (5 cm × 2.5 cm). Approximately 2 inches of masking tape was affixed to the edges of the card stock. The cloth and card stock were then placed on a drying rack and allowed to dry for at least 3 min prior to testing.

A test assay consisted of protecting the hand of each participant with a powder-free latex glove (Diamond Grip, Microflex Corporation, Reno, NV, USA). The gloved hand and arm were then placed inside a knee-high stocking (Leggs everyday knee highs, Winston-Salem, NC, USA). A plastic sleeve constructed of polyvinyl was then placed over the arm and stocking. The sleeve has a lengthwise Velcro seam to allow sealing over the arm. There was a window cut into the sleeve (4 × 8 cm opening) approximately half way between the wrist and elbow. This window allowed attractive odors from the skin surface to escape from the sleeve through the opening. The opening was covered with the treated cloth. The patch test order was randomized among volunteers and day-to-day.

The arm, sleeve and cloth were inserted into the mosquito cage for a 1 min period to determine if the compound and dosage on cloth were repellent. The number of feeding mosquitoes was determined by giving the arm a brisk shake after 1 min and counting the number of mosquitoes that remained biting through the cloth. This procedure was repeated with lower concentrations of the compound until the cloth failed to prevent a threshold level of bites. The failure point for these experiments was selected to be 5 bites (1% of the cage population biting). During the testing process, no more than 10 treatments were assayed in succession with a caged population of test mosquitoes before allowing a 15 min recovery period. This was necessary because upon repeated repellent exposure, mosquitoes fatigue and exhibit decreased response to attractant (skin) odors.

Four volunteers (three males and one female) participated in the studies of minimum effective dosage (MED) of the oils; three volunteers (all male) tested the single compounds. A series of dosages were used to evaluate the MED [43]. This series was based on mass such that the highest application rate was 1.5 mg/cm^2^ on cloth. The series then consisted of cloth treated at successively lower dilutions: 0.750, 0.375, 0.188, 0.094, 0.047, 0.023, and 0.011 mg/cm^2^. In some cases, there was not enough material to evaluate the higher concentrations; in this case the highest concentration was tested and successively lower concentrations are tested if the compound is found to be repellent. Testing for MED was initiated at the median concentration (typically 0.375 mg/cm^2^) on treated cloth and then by evaluating higher or lower treatment concentrations as necessary until all subjects had evaluated the cloths and pinpointed the concentration that produced the 1% (5 bites) failure point. If the 1.500 mg/cm^2^ (or highest concentration) on cloth was not efficacious (>5 bites in one minute), then the MED was noted as ineffective at the highest concentration tested. During a test, all volunteers wore a particular patch and tested for 1 min intervals. Patches were rotated among the volunteers, thus, no patch was evaluated beyond 10 min after the 3 min drying period to avoid any bias that may result from evaporative loss of treatment of the cloth during the duration of the test. All subjects provided informed consent and procedures were followed in direct accordance with those approved by the University of Florida Human Use Institutional Review Board-01 (Study # 636-2005). Essential oil standards (1,8-cineole, (−)-β-pinene, (−)-linalool, (+)-terpinen-4-ol, (−)-terpinen-4-ol, α-terpineol and β-bisabolol) (>95%) were purchased from Aldrich-Sigma (St. Louis, MO, USA).

### 3.7. Mosquito Larvicidal Bioassays

All essential oils were diluted in dimethyl sulfoxide (DMSO) and serial dilutions were performed for each test compound (seven concentrations between 8 and 500 mg/L). Larvae assays were performed in 24-well plates using 5 first instar-larvae in each well. Each well contained 950 μL of water, 40 μL of larvae food solution, and 10 μL of DMSO (control) or 10 μL of serially diluted test samples. Mortality data were recorded twenty-four hours post-exposure [27].

### 3.8. Mosquito Adult Topical Bioassays

To determine the toxicity of each chemical against female *Ae. aegypti*, the compound was serially diluted in acetone and topically applied to individual mosquitoes. Prior to topical application, 5 to 7 day-old females were briefly anaesthetized for 30 s with carbon dioxide and placed on a 4 °C chill table. A droplet of 0.5 μL of chemical solution was applied to the dorsal thorax using a 700 series syringe and a PB600 repeating dispenser. A screening dose of 3.125 mg/L per female was used on 25–30 females. Tests were replicated three times. If no mortality was found at this dose, further testing was not warranted. Control treatments with 0.5 μL of acetone alone gave mortality with less than 10%. After treatment, mosquitoes were kept in plastic cups and supplied with 10% sucrose solution for 24 h before mortality was recorded. Temperature and humidity were maintained at 26 °C and 80% RH, respectively [44].

### 3.9. Fire Ant Bioassays

Three red imported fire ant colonies were collected on February 26, 2009 in Sharkey County, MS, USA. Colony collection, separation, and maintenance followed the method developed by Banks *et al.* [45] and modified by Chen and Wei [46]. Both distilled water and 10% sugar water solution were provided in separate test tubes (2 × 15 cm). Each test tube was plugged with a cotton ball which served as feeding platform for the fire ants. In addition to 10% sugar water, house cricket, *Acheta domesticus*, was used as a food source. Petri dishes (14.0 cm × 2.0 cm) were used as artificial nests. On the bottom of the nest was 1.0 cm hardened dental plaster (Castone^®^, Dentsply International Inc. York, PA, USA). At the center of the nest was a 5.0-cm diameter brood chamber. There were two 8-mm access holes on the wall of the nest above the dental plaster. The lid of the nest was painted black (1302 Gloss Black Spray Enamel, Louisville, KY, USA) to block the light. All colonies were maintained at 25–30 °C.

The repellency was evaluated using a two-choice digging bioassay [47]. This method was based on the fact that a group of worker ants will always express digging behavior whenever an adequate digging substrate is available. Ants would not dig or would dig less in substrate containing a repellent. So the repellency in this study was defined as a suppression of ant digging behavior. The bioassay apparatus and sample preparation for digging bioassay were described by Chen *et al.* [48]. Briefly, four 2-mL centrifuge tubes were mounted under a 8.7 cm × 2.3 cm Petri dish using glue (Arrow Fastener Co., Inc., Saddle Brook, NJ, USA). Two tubes were filled with sand: one with treated sand and the other with control sand. The other two tubes were merely used to support the Petri dish. Two tubes with sand were 3.0 cm away from each other, located on a straight line that went through the center of the Petri dish, and at equal distance from the center of the Petri dish. There was a 3-mm diameter access hole, which went through the bottom of the Petri dish and the cap of the tube. The inner side of the Petri dish was coated with Fluon. Sand (Premium Play Sand, Plassein International, Longview, TX, USA) was first sieved through a #35 U.S.A. standard testing sieve (Thomas Scientific, Swedesboro, NJ, USA) and then washed with distilled water and dried at 350 °C for 2 h. The treated sand was prepared by mixing hexane/acetone solution with sand in an aluminum pan under a fume hood. Sand in the control tube was treated only with solvent. After solvent evaporated, distilled water was mixed with the sand to adjust the moisture content of the sand to 8%. In each tube a mean ± SD (2.86 ± 0.08 g) wet sand was added. Twenty fire ant workers were introduced into the center of the Petri dish. The experiment was conducted at room temperature. After 24 h, the sand in each vial was collected, dried at 250 °C for at least 4 h, and weighed. Information on concentrations, number of ant colonies and number of replicates for each bioassay are shown in Supplementary Table S2.

For each concentration of extract or compound, digging suppression index (DSI) was calculated using the formula DSI = (A_c_ − A_t_)/(A_c_ + A_t_), where A_c_ and A_t_ are the amounts of sand removed from control tube and treatment tube respectively. For each concentration, a paired *t*-test was used to compare mean amount of removed sand between treatment and control. The paired *t*-test was conducted using the pooled data from 2 to 3 colonies. 

## 4. Conclusions

The present study analyzed the essential oils of 19 *Hedychium* species and cultivars for their chemical compositions. For the most part, this is the first such report on these genotypes. The dominant components of the oils were monoterpenes and sesquiterpenes, especially 1,8-cineole (0.1%–42%), linalool (<0.1%–56%), α-pinene (3%–17%), β-pinene (4%–31%), and (*E*)-nerolidol (0.1%–20%). Self-sterility is common in *Hedychium* [49,50,51] so species of this genus readily hybridize, thus contributing to the current taxonomic confusion of *Hedychium* [52]. A few studies using molecular markers have been undertaken to determine *Hedychium* phylogeny [3,52]. Furthermore, Cole *et al.* [53] suggested that differences in chemical compositions of essential oils may provide useful characters in understanding phylogenetic relationships in species difficult to classify. Therefore, the results obtained in this study, in conjunction with morphological and genetic characteristics, could helpful in shedding more light on *Hedychium* taxonomy. The examined *Hedychium* essential oils showed significant insecticidal activities against azalea lace bugs and, to a lesser extent, against mosquitoes and fire ants, so these oils can potentially be used as biopesticides. Furthermore, *Hedychium* essential oils were ineffective against the fungi *Colletotrichum gloeosporioides*, *C. fragariae*, and *C. acutatum* in this study, but essential oils from the same plant materials totally inhibited the growth of pre-germinated spores of two other fungi, *Aspergillus flavus* and *Fusarium verticillioides* [54].

## Supplementary Materials

Supplementary materials can be accessed at: http://www.mdpi.com/1420-3049/18/4/4308/s1.

## References

[1] He E.Y. (2000). Study on *Hedychium coronarium* Koenig’s edibility and its pharmacological experiments. LishizhenMed. Res..

[2] Gopanraj G., Dan M., Shiburaj S., Sethuraman M.G., George V. (2005). Chemical composition and antibacterial activity of the rhizome oil of *Hedychium larsenii*. Acta Pharm..

[3] Gao L., Liu N., Huang B., Hu X. (2008). Phylogenetic analysis and genetic mapping of Chinese *Hedychium* using SRAP markers. Sci. Hort..

[4] Aqil F., Ahamad I. (2003). Broad-spectrum antibacterial and antifungal properties of certain traditionally used Indian medicinal plants. World J. Microbiol. Biotechnol..

[5] Rosa J.S., Mascarenhas C., Oliveira L., Teixeira T., Barreto M.C., Medeiros J. (2010). Biological activity of essential oils from seven Azorean plants against *Pseudaletia unipucta* (Lepidoptera: Noctuida). J. Appl. Entomol..

[6] Meazza G., Dayan F.E., Wedge D.E. (2003). Activity of quinones against *Colletotrichum* species. J. Agric. Food Chem..

[7] Curry K.J., Abril M., Avant J.B., Smith B.J. (2002). Strawberry anthracnose: Histopathology of *Colletotrichum acutatum* and *C. fragariae*. Mycology.

[8] Wedge D.E., Tabanca N., Sampson B.J., Werle C., Demirci B., Baser K.H.C., Nan P., Duan J., Liu Z. (2009). Antifungal and insecticidal activity from two *Juniperus* essential oils. Nat.Prod.Commun..

[9] Tabanca N., Bernier U.R., Tsikola M., Becnel J.J., Sampson B.J., Werle C., Demirci B., Baser K.H.C., Blythe E.K., Pounders C. (2010). *Eupatorium capillifolium* essential oil: Chemical composition, antifungal activity, and insecticidal activity. Nat.Prod.Commun..

[10] Carroll J.F., Tabanca T., Kramer M., Elajalde N.M., Wedge D.E., Bernier U.R., Coy M., Becnel J.J., Demirci B., Baser K.H.C. (2011). Essential oils of *Cupressus funebris*, *Juniperus communis*, and *J. chinensis* (Cupressaceae) as repellents against ticks (Acari: Ixodidae) and mosquitoes (Diptera: Culicidae) and as toxicants against mosquitoes. J.Vector Ecol..

[11] Lard C.F., Schmidt J., Morris B., Estes L., Ryan C., Bergquist D. (2006). An Economic Impact of Imported Fire Ants in the United States of America.

[12] Sampson B.J., McLaughlin J.L., Wedge D.E. (2003). Pawpaw extract as a botanical insecticide. Arthropod Manag.Tests.

[13] Sampson B.J., Tabanca N., Kirimer N., Demirci B., Baser K.H.C., Khan I.A., Spiers J.M., Wedge D.E. (2005). Insecticidal activity of 23 essential oils and their major compounds against adult *Aphis pseudobrassicae* (Davis) (Aphididae: Homoptera). Pest Manag. Sci..

[14] Weyerstahl P., Marschall H., Schneider S., Subba G.C. (1995). Constituents of the essential oil from the rhizomes of *Hedychium acuminatum* Roscoe. Flav. Fragr. J..

[15] Weyerstahl P., Marschall H., Thefeld K., Subba G.C. (1998). Constituents of the essential oil from the rhizomes of *Hedychium gardnerianum* Roscoe. Flav. Fragr. J..

[16] Medeiros J.R., Campos L.B., Mendonca S.C., Davin L.B., Lewis N.G. (2003). Composition and antimicrobial activity of the essential oils from invasive species of the Azores, *Hedychium gardnerianum* and *Pittosporum undulatum*. Phytochemistry.

[17] Bisht G.S., Awasthi A.K., Dhole T.N. (2006). Antimicrobial activity of *Hedychiums picatum*. Phytother. Res..

[18] Moellenbeck S., Koenig T., Schreier P., Schwab W., Rajaonarivony J., Ranarivelo L. (1997). Chemical composition and analyses of enantiomers of essential oils from Madagascar. Flav. Fragr. J..

[19] Gurib-Fakim A., Maudarbaccus N., Leach D., Doimo L., Wohlmuth H. (2002). Essential oil composition of Zingiberaceae species from Mauritius. J. Essential Oil Res..

[20] Zheljazkov V., Cantrell C.L., Tekwani B., Khan S.I. (2008). Content, composition, and bioactivity of the essential of three basil genotypes as a function of harvesting. J. Agric. Food Chem..

[21] Sangwan N.S., Farroqi A.H.A., Shabih F., Sangwan R.S. (2001). Regulation of essential oil production in plants. Plant Growth Regul..

[22] Argyropoulou C., Daferera D., Tarantilis P.A., Fasseas C., Polissiou M. (2007). Chemical composition of the essential oil from leaves of *Lippia citriodora* H.B.K. (Verbenaceae) at two developmental stages. Biochem. Syst. Ecol..

[23] Branney T.M.E. (2005). Hardy Gingers: Including Hedychium,Roscoea,and Zingiber.

[24] Kline D.L., Bernier U.R., Posey K.H., Barnard D.R. (2003). Olfactometric evaluation of spatial repellents for *Aedes aegypti*. J.Med. Entomol..

[25] Bernier U.R., Furman K.D., Kline D.L., Allan S.A., Barnard D.R. (2005). Comparison of contact and spatial repellency of catnip oil and *N*,*N*-diethyl-3-methylbenzamide (deet) against mosquitoes. J. Med. Entomol..

[26] Syed Z., Leal W.S. (2008). Mosquitoes smell and avoid the insect repellent DEET. Proc. Natl. Acad. Sci.USA.

[27] Pridgeon J.W., Becnel J.J., Clark G.G., Linthicum K.J. (2009). A high throughput screening method to identify potential pesticides for mosquito control. J. Med. Entomol..

[28] Anderson J.T., Thorvilson G.H., Russell S.A. (2002). Landscape materials as repellents of red imported fire ants. Southwest Entomol..

[29] Appel A.G., Gehret M.J., Tanley M.J. (2004). Repellency and toxicity of mint oil granules to red imported fire ants (Hymenoptera: Formicidae). J. Econ. Entomol..

[30] Chen J. (2009). Repellency of an over-the-counter essential oil product from China against workers of red imported fire ants. J. Agric. Food Chem..

[31] Curvers J., Rijks J., Cramers C., Knauss K., Larson P. (1985). Temperature programmed retention indexes: Calculation from isothermal data. Part 1: Theory. J. High Resolut. Chromatogr.

[32] Wang T., Sun Y. (1987). Definitions and methods of calculation of the temperature-programmed retention index, ITP. I. The relationship between ITP and the net retention volume for *n*-alkanes. J. Chromatogr. A.

[33] Adams R.P. (2001). Identification of Essential Oils Components by Gas Chromatography/Quadrupole Mass Spectroscopy.

[34] McLafferty F.W., Stauffer D.B. (1989). The Wiley/NBS Registry of Mass Spectral Data.

[35] Joulain D., König W.A., Hochmuth D.H. (2001). Terpenoids and Related Constituents of Essential Oils.

[36] Jennings W.G., Shibamoto T. (1980). Quantitative analysis of Flavor and FragranceVolatiles by Glass Capillary GC.

[37] Joulain D., König W.A. (1998). The Atlas of Spectra Data of Sesquiterpene Hydrocarbons.

[38] (1999). ESO 2000. The Complete Database of Essential Oils.Boelens Aroma ChemicalInformation Service.

[39] Wedge D.E., Klun J.A., Tabanca N., Demirci B., Ozek T., Baser K.H.C., Liu Z., Zhang S., Cantell C.L., Zhang J. (2009). Bioactivity-guided fractionation and GC/MS fingerprinting of *Angelica sinensis* and *Angelica archangelica* root components for antifungal and mosquito deterrent activity. J. Agric. Food Chem..

[40] Ramallo I.A., Zacchino S.A., Furlan R.L.E. (2006). A rapid TLC autographic method for the detection of xanthine oxidase inhibitors and superoxide scavengers. Phytochem. Anal..

[41] Posey K.H., Schreck C.E. (1981). An airflow apparatus for selecting female mosquitoes for use in repellent and attraction studies. Mos News.

[42] Barnard D.R., Bernier U.R., Xue R.D., Debboun M., Debboun M., Frances S.P., Strickman D. (2007). Standard Methods for Testing Mosquito Repellents. Insect Repellents Principles Methods,Uses.

[43] (1977). Walter Reed Army Institute of Research. RepellentActivity of Compounds. Part I. Protection Time and Minimum Effective Dosage against Aedes aegypti Mosquitoes.

[44] Pridgeon J.W., Pereira R.M., Becnel J.J., Allan S.A., Clark G.G., Linthicum K.J. (2008). Susceptibility of *Aedes aegypti*, *Culex quinquefasciatus* Say, and *Anopheles quadrimaculatus* Say to 19 pesticides with different modes of action. J. Med. Entomol..

[45] Banks W.A., Lofgren C.S., Jouvenaz D.P., Stringer C.E., Bishop P.M., Williams D.F., Wojcik D.P., Glancey B.M. (1981). Techniques for Collecting,Rearing,and Handling Imported Fire Ants.

[46] Chen J., Wei X. An Improved Method for Fast and Efficient Fire Ant Colony Separation,2005. Proceedings of Annual Red Imported Fire Ant Conference.

[47] Chen J., Cantrell C.L., Duke S.O., Allen M.L. (2008). Repellency of callicarpenal and intermedeol against workers of imported fire ants. J. Econ. Entomol..

[48] Chen S.S., Liu J.Y., Lin C.Y., Hsui Y.R., Lu M.C., Wu W.J., Chang S.T. (2008). Terminating red imported fire ants using *Cinnamonum osmpophloeum* leaf essential oil. Bioresource Technol..

[49] Holttum R.E. (1950). The Zingiberaceae of the Malay Peninsula. Garden’s Bull. Singapore.

[50] Gao J.Y., Ren P.Y., Li Q.J. (2005). Advances in the study of breeding system and pollination biology of gingers (Zingiberaceae and Costaceae). Acta Phytotaxon. Sin..

[51] Sakhanokho H.F., Rajasekaran K. (2010). Pollen biology of ornamental ginger (*Hedychium* spp. J. Koenig). Sci. Hort..

[52] Wood T.H., Whitten W.M., Williams N.H. (2000). Phylogeny of *Hedychium* and related genera (Zingiberaceae) based on ITS sequence data. Edinburgh J. Bot..

[53] Cole R.A., Haber W.A., Setzer W.N. (2007). Chemical composition of essential oils of seven species of *Eugenia* from Monterverde, Costa Rica. Biochem. Syst. Ecol..

[54] Rajasekaran K., Sakhanokho H.F., Tabanca N. (2012). Antifungal activities of *Hedychium* essential oils and plant extracts against mycotoxigenic fungi. J. Crop Improv..

