# Supplementary material for: Chemical Composition, Antifungal and Insecticidal Activities of Hedychium Essential Oils
Source: Molecules. 2013 Apr 11;18(4):4308–27. doi: 10.3390/molecules18044308 (PMC6270349; doi:10.3390/molecules18044308)
Supplement: Supplementary file 1 [file molecules-18-04308-s002.pdf]

Supplementary Materials

Table S1. Mean (SE) weight (g) of sand removed by worker ants 24 h after they were released in the two choice digging bioassay.

		Sand removed (SE)				
**Chemical**	**Concentration (mg/kg)**	**Treatment**	**Control**	**Digging suppression index (SE)**	**t-Value**	***p*-Value**
Tai Conch Pink	1	0.48 (0.037)	0.39 (0.045)	−0.15 (0.078)	−1.43	0.17
	10	0.49 (0.037)	0.51 (0.035)	0.04 (0.053)	0.49	0.6271
	100	0.47 (0.051)	0.55 (0.052)	0.08 (0.094)	1.03	0.3127
H. thyrsiforme	1	0.62 (0.054)	0.59 (0.070)	−0.07 (0.078)	−0.31	0.7569
	10	0.66 (0.059)	0.63 (0.062)	−0.03 (0.070)	−0.37	0.7168
	100	0.46 (0.052)	0.62 (0.056)	0.16 (0.090)	2.25	0.0321
Dave Cave	1	0.74 (0.067)	0.43 (0.050)	−0.31 (0.050)	−5.32	<0.0001
	10	0.77 (0.056)	0.56 (0.058)	−0.20 (0.046)	−4.27	0.0002
	100	0.82 (0.066)	0.56 (0.061)	−0.24 (0.047)	−5.17	<0.0001
Pink V	1	0.57 (0.042)	0.46 (0.043)	−0.16 (0.052)	−3.17	0.0036
	10	0.63 (0.053)	0.45 (0.040)	−0.19 (0.042)	−5.03	<0.0001
	100	0.61 (0.046)	0.53 (0.054)	−0.12 (0.060)	−1.46	0.1546
White Starburst	1	0.52 (0.050)	0.32 (0.048)	−0.23 (0.103)	−2.87	0.0076
	10	0.57 (0.071)	0.39 (0.045)	−0.22 (0.090)	−2.57	0.0155
	100	0.48 (0.051)	0.49 (0.061)	−0.11 (0.102)	0.22	0.822
H. elatum	1	0.36 (0.039)	0.23 (0.040)	−0.34 (0.067)	−4.14	0.003
	10	0.39 (0.041)	0.28 (0.038)	−0.20 (0.0716)	−3.1	0.0043
	100	0.38 (0.040)	0.34 (0.040)	−0.05 (0.069)	−0.8	0.4325
Dr. Moy	1	0.33 (0.034)	0.56 (0.054)	0.19 (0.091)	3.19	0.0034
	10	0.26 (0.045)	0.40 (0.045)	0.22 (0.070)	2.91	0.0069
	100	0.35 (0.045)	0.41 (0.049)	0.08 (0.052)	1.99	0.0561
Pink Sparks	1	0.48 (0.059)	0.71 (0.049)	0.21 (0.075)	2.9	0.0146
	10	0.41 (0.030)	0.62 (0.056)	0.19 (0.059)	3.81	0.0029
	100	0.38 (0.052)	0.67 (0.048)	0.31 (0.083)	5.5	0.0002
H. flavum	1	0.45 (0.032)	0.43 90.045)	−0.03 (0.062)	−0.26	0.7963
	10	0.66 (0.076)	0.54 (0.064)	−0.10 (0.073)	−1.45	0.1759
	100	0.83 (0.055)	0.65 (0.040)	−0.16 (0.038)	−3.29	0.0072

Table S1. *Cont.*

		Sand removed (SE)				
**Chemical**	**Concentration (mg/kg)**	**Treatment**	**Control**	**Digging suppression index (SE)**	**t-Value**	***p*-Value**
	1	0.45 (0.067)	0.33 (0.045)	−0.14 (0.097)	−1.81	0.0097
H. bousigonianum	10	0.61 (0.051)	0.31 (0.043)	−0.36 (0.070)	−6.5	<0.0001
	100	0.43 (0.058)	0.32 (0.082)	−0.25 (0.137)	−0.97	0.3514
	1	0.55 (0.060)	0.51 (0.063)	−0.04 (0.105)	−0.4	0.6959
Tai Monarch	10	0.48 (0.069)	0.65 (0.052)	0.17 (0.075)	2.16	0.0538
	100	0.61 (0.047)	0.57 (0.051)	−0.04 (0.075)	−0.43	0.6759
	1	0.43 (0.060)	0.30 (0.053)	−0.20 (0.121)	−1.81	0.0972
Tai Empress	10	0.51 (0.045)	0.33 (0.043)	−0.21 (0.074)	−3.11	0.0099
	100	0.53 (0.030)	0.27 (0.034)	−0.35 (0.071)	−6.49	<0.0001
	1	0.53 (0.053)	0.34 (0.048)	−0.26 (0.077)	−3.79	0.003
Tai Emperor	10	0.44 (0.056)	0.37 (0.055)	−0.85 (0.145)	−0.69	0.5066
	100	0.40 (0.052)	0.38 (0.052)	−0.01 (0.128)	−0.2	0.8478
	1	0.42 (0.044)	0.27 (0.037)	−0.23 (0.037)	−4395	0.0004
H. forresti	10	0.34 (0.050)	0.23 (0.053)	−0.26 (0.174)	−1.36	0.2022
	100	0.44 (0.054)	0.23 (0.042)	−0.30 (0.157)	−2.57	0.0262
	1	0.37 (0.040)	0.22 (0.049)	−0.32 (0.121)	−2.6	0.0246
H. coccineum	10	0.35 (0.051)	0.34 (0.048)	−0.05 (0.131)	−0.19	0.8534
	100	0.30 (0.048)	0.30 (0.045)	0.02 (0.125)	0.03	0.9771
	1	0.30 (0.033)	0.42 (0.029)	0.25 (0.062)	3.88	0.0025
Kinkaku	10	0.33 (0.045)	0.39 (0.055)	0.07 (0.102)	0.95	0.3639
	100	0.25 (0.046)	0.35 (0.047)	0.21 (0.117)	1.75	0.1074
	1	0.49 (0.053)	0.47 (0.041)	−0.005 (0.067)	−0.23	0.8256
Tai Mammoth	10	0.49 (0.055)	0.32 (0.047)	−0.20 (0.113)	−1.97	0.0749
	100	0.50 (0.042)	0.48 (0.069)	−0.05 (0.076)	−0.27	0.7934
H. flavescens	1	0.37 (0.049)	0.25 (0.054)	−0.21 (0.140)	−1.36	0.1996

Table S2. Concentrations, number of colonies, and number of replicates for each bioassay.

Chemical	Concentration (mg/kg)	Number of colonies	Replicates per bioassay
Tai Conc Pink	1.0, 10.0, 100.0	3	10
H. thyrsiforme	1.0, 10.0, 100.0	3	10
Dave Case	1.0, 10.0, 100.0	3	10
Pink V	1.0, 10.0, 100.0	3	10
White Starburst	1.0, 10.0, 100.0	3	10
H. elatum	1.0, 10.0, 100.0	3	10
Dr. Moy	1.0, 10.0, 100.0	3	10
Pink Sparks	1.0, 10.0, 100.0	3	6
H. flavum	1.0, 10.0, 100.0	3	6
H. bousigonianum	1.0, 10.0, 100.0	3	6
Tai Monarch	1.0, 10.0, 100.0	3	6
Tai Empress	1.0, 10.0, 100.0	3	6
H. forrestii	1.0, 10.0, 100.0	3	6
H. coccineum	1.0, 10.0, 100.0	3	6
Kinkaku	1.0, 10.0, 100.0	3	6
Tai Mammoth	1.0, 10.0, 100.0	3	6
H. flavesens	1.0, 10.0, 100.0	3	6
